# Dialysis decisions concerning cognitively impaired adults: a scoping literature review

**DOI:** 10.1186/s12910-021-00591-w

**Published:** 2021-03-05

**Authors:** Jordan A. Parsons, Jonathan Ives

**Affiliations:** Centre for Ethics in Medicine, Bristol Medical School, University of Bristol, Bristol, UK

**Keywords:** Chronic kidney disease, Cognitive impairment, Dementia, Dialysis, Kidney failure, Mental Capacity Act 2005

## Abstract

**Background:**

Chronic kidney disease is a significant cause of global deaths. Those who progress to end-stage kidney disease often commence dialysis as a life-extending treatment. For cognitively impaired patients, the decision as to whether they commence dialysis will fall to someone else. This scoping review was conducted to map existing literature pertaining to how decisions about dialysis are and should be made with, for, and on behalf of adult patients who lack decision-making capacity. In doing so, it forms the basis of a larger body of work that is exploring how these decisions ought to be made.

**Methods:**

To identify relevant papers, searches were conducted on Ovid MEDLINE(R), Embase, PsychINFO, The Cochrane Library, and Web of Science. Inclusion criteria were then applied, requiring that papers: report on empirical studies about how decisions about dialysis are made *and/or* discuss how decisions about dialysis should be made with, for, and on behalf of adult patients who lack decision-making capacity; be published from 1961 onwards; and be published in English. This resulted in 27 papers eligible for inclusion.

**Results:**

Of note, the majority of papers originated in the United States. There was wide variation across the included papers. Extracted data were grouped under the following themes: involving various parties (patient involvement, family dominance, and wider communication); objectivity about care options (including difficulties with family detachment); cultural sensitivity; medical versus non-medical factors; managing nonadherent patients; and the role and prevalence of substituted judgement. The literature shows that there is inconsistency in the principles and processes surrounding decisions made about dialysis with, for, and on behalf of adult patients who lack decision-making capacity.

**Conclusions:**

This scoping review demonstrates that there is significant variation in both the practice and theory of dialysis decision making with, for, and on behalf of cognitively impaired adult patients. Complexity arises in considering who should get a say, how influential their say should be in a decision, and what factors are most relevant to the decision. A lack of up-to-date literature exploring this issue is highlighted, with this scoping review providing a useful groundwork from which further research can be undertaken.

## Background

End stage kidney disease (ESKD, or kidney failure) affects a significant proportion of the world’s population. Chronic kidney disease (the precursor to ESKD) rose from being the 27th highest cause of global deaths in 1990 to 18th in 2010 in the Global Burden of Disease study (p. 2113) [1]. Several options exist for the care of patients with or approaching ESKD, including transplantation, dialysis, and conservative kidney management (CKM). When transplantation is an option but cannot happen immediately, dialysis often acts as a bridge therapy. When transplantation is not an option, the decision must be made as to whether to dialyze the patient at all. If yes, the patient will receive a whole package of care including dialysis. If not, the patient will receive CKM, which is a similar care plan only without dialysis. As such, many patients with ESKD find themselves on dialysis at some point. Of these, a not insignificant proportion contemporaneously suffer cognitive impairment [2]. This is, in part, due to an ageing population, as ESKD is often reached in later life. In addition, vascular comorbidities have been found to be a major contributing factor to cognitive decline [3, 4].

Patients *with* decision-making capacity in relation to dialysis may choose to forego dialysis in favour of CKM, for reasons that often relate to the relationship between quality of life and the burden of dialysis. One study reported patients choosing to forego dialysis because of its ‘arduous nature’ and related practical reasons such as attending the hospital three times each week [5]. Assuming these decisions are informed and voluntary, they seem to be valid reasons why a patient with or approaching ESKD might not want to commence dialysis. Others will make different decisions about the balance of benefits and burdens, based on different life goals which, accepting the need to respect autonomy, are equally legitimate. That being the case, it seems very likely that among patients who lack the necessary decision-making capacity there will be a range of personal contexts, views, and preferences that would, in the absence of cognitive impairment, lead to a range of different decisions about dialysis being made.

Given that, it is important to reflect on how dialysis decisions should be made with, for, and on behalf of persons who lack capacity. This paper reports the first (mapping [6]) stage in a larger project exploring how decisions about maintenance dialysis ought to be made when the patient is cognitively impaired, within the context of the Mental Capacity Act 2005 (MCA 2005) in England and Wales. This first stage aimed to map what is currently known about how and why decisions about maintenance dialysis are made for persons lacking capacity, with a view to informing the later normative work. The focus is on adults (18 +), as the MCA 2005 does not apply to minors.^1^ Under the MCA 2005, in the absence of a formally appointed surrogate decision maker, section 1(5) requires that a decision be made in the patient’s ‘best interests’. Section 4 of the MCA 2005 provides a checklist of factors to consider in determining the patient’s best interests, though is argued by some to be merely enumerative and to lack sufficient guidance on the weighting of these considerations [7]. Nonetheless, the MCA 2005 best interests approach is framed as being distinct from a substituted judgement approach. Whereas substituted judgement requires the substitute decision maker to ascertain and make the decision the patient would have made if capacitous, best interests decisions, in theory at least, require the decision maker only to *consider* the decision the patient would have made among several factors, and to actually make the decision that best accords with the patient’s previously expressed values and beliefs. Essentially, a best interests decision is to be the best decision that the patient *could* make, not necessarily the decision they *would* have made.^2^

## Methods

The project team (JAP and JI) utilised the five key stages of Arksey and O’Malley’s six stage process for scoping reviews: (1) identifying the research question; (2) identifying relevant papers^3^; (3) paper selection; (4) charting the data; and (5) collating, summarizing, and reporting the results [8]. The sixth stage—consultation with practitioners/consumers—is an optional stage. As this scoping review is part of a larger project, the sixth stage will be carried out later in the project.

### Stage 1: identifying the research question

The research question was initially developed by JAP following informal discussions with several nephrologists and ethicists and finalised in conjunction with JI. The primary research question settled on was ‘What is known and theorised about how decisions about maintenance dialysis are and should be made with, for, and on behalf of adult patients who lack decision-making capacity?’. In answering this question, this scoping review maps evidence pertaining to three secondary research questions: (1) how are decisions about maintenance dialysis for adult patients who lack decision-making capacity made in practice?; (2) how do different stakeholders understand the process of making decisions about maintenance dialysis for adult patients who lack decision-making capacity?; and (3) what normative arguments exist concerning how decisions about maintenance dialysis should be made with, for, and on behalf of adult patients who lack decision-making capacity?

### Stage 2: identifying relevant papers

Relevant papers were identified by searching several research databases. A preliminary search was carried out in February 2020 by JAP using Ovid Medline. This search was conducted with reference to the research questions already detailed, and with assistance from a medical librarian. The purpose of this search was to identify search terms, abbreviations, and Medical Subject Headings (MeSH) terms used frequently in the area with which the research questions are concerned. Following this search, the final search string was decided on (see Table 1).^4^

**Table 1:** Final search string

Facet 1		Facet 2		Facet 3
exp Decision Making (MeSH) **OR** exp Clinical Decision-Making (MeSH) **OR** substituted judg?ment.tw **OR** surrogate decision maker.tw **OR** best interest*.tw	**AND**	exp Mental Competency (MeSH) **OR** exp Dementia (MeSH) **OR** mentally incapacitated patient*.tw **OR** mental capacity act.tw **OR** cognitively impaired.tw **OR** cognitive impairment.tw	**AND**	exp Renal Dialysis (MeSH) **OR** dialysis.tw **OR** kidney failure.tw **OR** renal failure.tw **OR** end-stage kidney disease.tw

Using this search string, the final search was carried out on 3rd March 2020 on five databases: Ovid MEDLINE(R), Embase, PsychINFO, The Cochrane Library, and Web of Science. These databases were selected based on their indexing of relevant journals. All databases were searched from 1961 (when maintenance dialysis was introduced in the UK) to the day of the search.^5^

#### Ancillary search strategies

In addition to online searches, the reference lists of papers included following the application of inclusion and exclusion criteria (see below) were hand searched for additional potentially relevant papers. Papers on reference lists were considered for relevance based on their titles, and those deemed likely to be suitable for inclusion were screened in full.

### Stage 3: paper selection

**Fig. 1:**
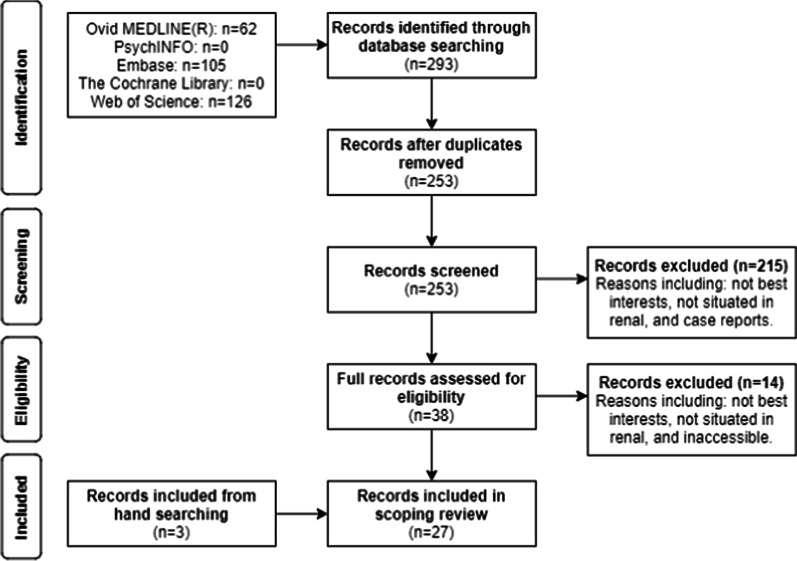
PRISMA diagram of paper identification

Once papers were identified as per Stage 2, they were subjected to a screening process to determine which papers would be included (see Fig. 1). Initially, all duplicate papers were removed. The titles and abstracts of all remaining papers were then screened by the first reviewer (JAP) according to pre-specified inclusion and exclusion criteria and classified as “include”, “exclude”, or “unsure”. The inclusion and exclusion criteria were as follows:

Inclusion:

- Papers, books, book chapters.
- Papers that report on empirical studies about how decisions about dialysis are made with, for, and on behalf of adult patients who lack decision-making capacity (this does not have to be the primary focus of the paper).
- Papers that discuss how decisions about dialysis should be made with, for, and on behalf of adult patients who lack decision-making capacity (this does not have to be the primary focus of the paper).
- Papers published since 1961 (the year in which maintenance dialysis was introduced in the UK).
- Papers published in English.

Exclusion:

- Grey literature, including journalism.
- Case reports.
- Papers not situated in renal care.
- Papers about paediatric or neonatal renal care.
- Papers that do not reference best interests or lack of capacity.
- Papers not in English.

A random sample of 10% of the “include” and “exclude” lists, as well as all of those classified as “unsure”, were screened by a second reviewer (JI). Whilst there was initial agreement on the classification of most papers, there were some discrepancies that necessitated discussion. Had discussions not resolved disagreements, a third reviewer would have reviewed the paper(s) in question and the majority decision carried; this did not prove necessary. Several papers did not have an abstract or had an abstract which did not allow for a decision as to its inclusion or exclusion to be made confidently. To ensure an accurate decision, the full texts of these papers were screened against the inclusion and exclusion criteria.

Following discussion with the second reviewer, papers on the “include” list, as well as those with no/unclear abstracts, were screened in full against the inclusion and exclusion criteria. 14 papers were excluded at this point, including one which could not be accessed (despite all reasonable efforts being made) [9]. 24 papers were identified as fitting the inclusion criteria and were included in the review.

Following the selection of included papers, the titles in all reference lists were screened to identify further papers that might have fitted the inclusion criteria. The screening process outlined above was then repeated for all additional papers identified in this way. Only three papers were found this way that were deemed suitable for inclusion.^6^

The final list of papers deemed to fit the inclusion criteria was then compiled for data extraction. This list totalled 27 papers (24 from database searches and three from ancillary searching), comprising a mixture of empirical and normative work.

### Stage 4: charting the data

Once a final list of papers to include was compiled, each was read through twice by the first reviewer (JAP), and data extracted on the second reading. The data were charted using Microsoft Excel based on the following paper characteristics:

i. Paper (author(s) and year of publication)
ii. Country of origin
iii. Aim(s) of paper
iv. Method(s)/study type
v. Results
vi. Reflections from reviewer^7^

To aid reliability, the second reviewer (JI) reviewed a sample of 10% of the papers.

### Stage 5: collating, summarizing, and reporting the results

An inductive, data-driven approach to analysing the charted data was taken, employing thematic analysis [10]. Thematic analysis is used for ‘analysing and reporting patterns’ (p. 79) in data [10], which are then described ‘in words rather than numbers’ (p. 380) [11]. As such, any language indicative of frequency ought not to be taken as a representation of significance. The data were not quantitatively analysed, and such language is used merely to provide a sense of those views and findings that were more or less prevalent in the papers.

Broadly, six main themes were identified: “involving various parties”; “objectivity”; “cultural sensitivity”; “medical versus non-medical factors”; “managing nonadherent patients”; and “substituted judgement”. Discrete sub-themes were developed within each broad theme—all of which are detailed in the next section.

## Results

Before detailing the results of this review, it is worth briefly noting the geography of the included papers. The wider concern of this project is with best interests as per the MCA 2005. Of note, however, very few included papers originated in England and Wales (or the wider UK). As demonstrated by Table 2, only five of 27 papers originated in the UK (of these, one was an international collaboration, and another concerned an Australian case). The US, on the other hand, accounted for 16 papers. This must be taken into account when reading the results, as a range of jurisdictions are represented and perspectives from the US represent the simple majority of papers.

**Table 2:** Countries of origin of included papers

Paper	Country/ies
Ang et al. [22]	Singapore
Brennan et al. [13]	Australia, Canada, United States, and United Kingdom
Cady [36]	United States
Campbell et al. [32]	United States
Clement et al. [21]	France
Conneen et al. [15]	United States
Davison and Holley [34]	Canada
DeCamp [23]	United States
Feely et al. [12]	United States
Foote et al. [25]	Australia and New Zealand
Grubb [38]	United Kingdom
Jones and McCullough [28]	United States
Kaye and Lella [16]	Canada
Keating et al. [17]	United States
MacPhail et al. [14]	Australia
McDougall [24]	United Kingdom (concerning an Australian case)
Moss et al. [27]	United States
Munoz Silva and Kjellstrand [29]	United States
O’Dowd et al. [30]	United States
O’Rourke et al. [37]	United Kingdom
Perkins [26]	United States
Pruchno et al. [18]	United States
Scott et al. [20]	United Kingdom
Sehgal et al. [35]	United States, Germany, and Japan
Spike [33]	United States
Spike [19]	United States
Ying et al. [31]	Canada

Totals: Singapore (n = 1) | New Zealand (n = 1) | France (n = 1) | Germany (n = 1) | Japan (n = 1) | Australia (n = 4) | Canada (n = 5) | United Kingdom (n = 5) | United States (n = 16)

Between these jurisdictions there are likely to be both significant and nuanced differences in the prevailing social and cultural values and norms that inform medical decision making, legislative frameworks, and how healthcare systems operate. This variation will inevitably affect how decisions are made with, for, and on behalf of adult patients who lack decision-making capacity, as such decisions are embedded within the systems in which they are made. As we will come to discuss, the concept of atomistic autonomy is more highly valued in some countries than in others, with some favouring a more communitarian approach—and this will be reflected in both the way that decisions are intuitively made and by the legislative, institutional, and professional frameworks within which such decisions have to be made. Even within a single country, of course, there will be cultural variation that can affect perceptions of the decision-making process in a healthcare setting. This latter issue will be highlighted as a minor, but nonetheless important, theme arising from the literature. Equally, the structure of healthcare systems is likely to play an important role, with some of the represented jurisdictions operating insurance-based healthcare and others operating some form of publicly funded system. This kind of difference, again, may explain variation in the way that decisions are made with, for, and on behalf of persons who lack decision-making capacity, even when prevailing values are shared. Whilst our aim in this paper is not to undertake comparative analysis of this kind, we will consider this as and when it becomes relevant to the discussion.

### Involving various parties

Perhaps the most prominent theme from the literature is the complexity of these decisions in terms of which parties are and/or ought to be involved. When the patient is unable to make an autonomous decision about their own care, who should be involved in making the decision for them—and especially what balance of influence is appropriate—is not straightforward. The primary focus of the literature in this regard is the role of cognitively impaired patients themselves and those close to them. However, there is also some discussion of interaction between professionals involved in the care of such patients.

#### Involving the patient

Even where the patient lacks the necessary mental capacity for dialysis decisions, the value of that patient being involved in decisions about their own care is highlighted. This is, in general, framed in terms of recognising the importance of patient autonomy, even though such patients would not be deemed capable of autonomous choice. Some papers consider a duty on nephrologists to prepare for situations in which a patient has lost decision-making capacity. However, it is noted that patient wishes are often unclear [12].

First, Brennan and colleagues note that dementia does not necessarily mean a patient lacks decision-making capacity [13], which reflects the principles of the MCA 2005. They argue that where capacity is uncertain, an assessment ought to be carried out. This is echoed by MacPhail and colleagues who assert the importance of recognising capacity as decision specific and that, therefore, patients with dementia (especially mild to moderate) may well be able to meaningfully participate in decisions about their own care [14].

There appears to be strong support for some form of care planning as a means of involving patients in decisions about their own care [14–19]. This is in part due to a recognition that a decline in a patient’s cognitive functioning may go unnoticed until a critical incident, at which point it is too late to involve them in the decision-making process [14], but also that making decisions may prove less traumatic when the patient’s own wishes are known [15]. In a study by Scott and colleagues [20], several nephrologists viewed advance care planning (ACP) as a way of avoiding the need for a best interests decision. However, other participants in the same study felt it inappropriate to always follow previously stated patient preferences as it may be hard for patients to anticipate how they will feel about different treatment options ahead of time and the change in their health status may affect their preferences [20]. This was also highlighted by Conneen and colleagues [15]. Further, Clement and colleagues found that only 58% of French nephrologists participating in their study said they would be influenced by a patient’s refusal to initiate dialysis, and that the majority would not respect a patient’s request to discontinue dialysis (whether of ‘sound mind’ or not) [21].

In anticipation of a patient being unable to make their own dialysis decision, Keating and colleagues note the importance of advance directives as a specific form of ACP, and argue that they should be respected as a reflection of patient wishes [17]. Similarly, Ang and colleagues raise the idea of Ulysses contracts^8^ as a means of capturing patients’ earlier stated preferences in case their cognition starts to decline [22]. However, DeCamp details a case in which the patient had written a living will, but the terms used were so general as to be practically useless [23]. It is important, then, according to some, for there to be good communication between patients and surrogate decision makers before the need for a decision to be made arises [16, 18]. Such communication, it is suggested, should consider the preferences of the patient at different points in the progression of their illnesses to understand when they might change their view; this should also involve the surrogate decision maker developing an understanding of the reasons *why* the patient has the preferences they do so that they can better make decisions (consistent with those reasons) if faced with a scenario that had not been discussed [18]. Kaye and Lella go on to argue for the documentation of these discussions, as well as regularly revisiting them [16]. MacPhail and colleagues also advocate for regular cognitive assessments to allow impairment to be discovered quickly [14].

However, Kaye and Lella also suggest that it can be appropriate to set aside the known wishes of the patient when doing so may benefit the family, going as far as to suggest it is mandatory unless the patient is undoubtedly experiencing severe suffering [16]. This point is made in response to the particular case they discuss, in which the family later wrote to clinical staff explaining that on reflection they recognise that their insistence on keeping their relative alive on dialysis was a result of them thinking of themselves. The family justified this, stating that they do not consider it wrong to have done so as ‘this helpless man was bringing out something good from all of us’ [16]. On this, Kaye and Lella also argue that the reverse can be true; if clinical staff and the family feel that the continuation of dialysis will cause unnecessary suffering, dialysis may be discontinued even if the patient had made clear that they wanted to continue [16]. This is an outlier insofar as it questions the underlying importance of the patient’s own preferences (which other scholars have deemed of great importance and to be followed if known [17]), and suggests that the interests of the family should be explicitly considered.^9^

It must be noted, however, that in practice patients may not be involved in decisions about their care to the degree that this literature suggests they should. Some papers noted that patients may be overlooked due to a perception that cognitive impairment precludes any informed decision making, in part because of the complexity of dialysis [14, 20]. Indeed, McDougall discusses a specific patient and notes that she cannot be involved in the decision due to her dementia [24]. Scott and colleagues found there to be divided appreciation of ACP [20], which seems to again suggest exclusion rather than inclusion of patient views. On balance, this may be in part explained by the fact that a patient’s ACP may show that they do not want dialysis if they develop dementia but provide insufficient details to act on; in practice, the preferences of patients vary between mild, moderate, and advanced dementia, with many wanting to forego dialysis only in the event of advanced dementia [14].

#### Family dominance

A particularly prominent sub-theme is that of family dominance in decision making. Whilst there is recognition that nephrologists are not obliged to provide treatment they consider inappropriate or excessively burdensome [13] and some nephrologists noted that they do not consider the family’s decisions to be of primary importance [25, 26], some of the included papers reported that a fear of complaints or litigation can lead nephrologists to agree to the demands of relatives even when they think them not to be in the patient’s best interests—so-called “defensive medicine” [12, 17, 22].^10^ Moss and colleagues found that 99% of dialysis unit medical directors surveyed would consult the family on care decisions concerning a patient who develops dementia [27]. In one US case, the clinical team agreed to the demands of an insistent family after being advised to do so by the hospital’s legal team, because the family were substantial donors to that hospital [28]. In another, the wishes of the patient were, in essence, overridden by his daughter; a 72-year-old male had received two kidney transplants and did not want to return to dialysis if the second failed, but when it did fail his daughter insisted and the patient finally agreed [13]. The line between persuasion and coercion was not clear in this case, and whilst this particular example concerns a patient who was able to agree himself, it is still demonstrative of the dominance of family in dialysis decisions, and it is reasonable to assume that this would be more pronounced in cases of cognitively impaired patients.

This represents a clear disparity between generally accepted theory and practice. Theory tells us that the patient comes first and that decisions ought to reflect what is best for the patient, accounting for what the patient would likely have wanted. In practice, there is evidence that suggests this often does not happen. It is noteworthy, when considering family dominance in decision making, that there is evidence of families frequently failing to reflect the choices the patient would have made—generally being more in favour of life-sustaining treatment [17, 18]. MacPhail and colleagues also note that families mostly choose dialysis, but also that they often report being uninformed and unprepared to make these decisions [14].

Whilst some argue that family should be involved in decisions [22]—generally on the basis that they will represent the interests of the patient [17]—there are equally concerns that substituted judgement (which is used in many legal jurisdictions as a way of making these decisions) may not be appropriate where the relative who is making the decisions would be the primary caregiver—especially in countries where the carer burden is significant [23]. Relatives may also be unsuitable proxies if they do not know the patient well [26]. This leads Keating and colleagues to argue that there is no moral authority for family to make medical decisions which do not reflect the patient’s wishes and, as such, nephrologists need not automatically comply with family decisions [17]—and this reflects the “best interests” system in England and Wales. Perkins similarly employs fiduciary principles to argue that the clinician is responsible for ensuring that any decision making adequately protects the interests of the cognitively impaired patient, which may mean making a decision without the input of the patient’s relatives [26].

Notwithstanding those difficulties and differences, there is broad consensus across the included papers that the family ought to be afforded *some* role in the decision-making process. Munoz Silva and Kjellstrand found there to be a trend in families taking a larger role in these decisions over time (having charted such decisions in the US from 1970 to 1983) [29]. This is perhaps unsurprising at a time when medical paternalism was coming into question; where patient autonomy is not possible, vesting the decision-making power in the family might be considered preferable to the judgement of the treating nephrologist.^11^

Arguably at one extreme, Kaye and Lella reason that where there is a ‘significant benefit to be gained by the family’—meaning some sort of solace or an opportunity for family members living far away to come and say goodbye—it would be ‘mandatory’ to override the previously stated wishes of the patient and keep them on dialysis (p. 78) [16]. Such a position places the interests of family on par with, or even above, those of the patient.

Others have advocated for positions that still generally favour the family being central in the decision-making process [15, 17, 30], but fall significantly short of giving preference to their interests.^12^ For example, O’Dowd and colleagues, reflecting on a case in which they had prevented the brother of an incapacitated patient acting as proxy decision maker despite the patient having previously expressed such a preference, describe how they later came to regret that decision [30]. They concluded that it would have been ‘better to go with the nonstranger surrogate than for us, who are all strangers, to make the decision’ (p. 324) [30]. Consulting the family extensively is similarly valued by Keating and colleagues, though they remain strongly supportive of the position that doctors are not obligated to provide any treatment they do not consider appropriate, and that there is no moral authority for families to make decisions independent of either the patients’ wishes or best interests [17]. They do, however, strongly imply the family is a very powerful actor who they would not want to go against, preferring to transfer a patient to another care provider^13^ rather than provide treatment against the family’s wishes. Before doing so, however, they advocate attempting to understand the reasons for the family’s position, as this may highlight an easily resolved misunderstanding and help find resolution [17]. It is noteworthy that Keating and colleagues maintain this position, which implicitly accepts a significant role for family, despite noting how studies have demonstrated that families frequently fail to reflect the choices the patient would have made [17].

Support for such a significant role for family is not, however, unanimous. McDougall writes:

‘Although there is generally a very important role for families in medical decision-making for incompetent patients, this role should be highly sensitive to the specific details of the patient’s situation and the nature of the particular family involved’ (p. 45) [24].

This position arises out of the case discussed by McDougall, in which she considers the view of the family to be wrong [24]. This led her to question the common assumption that the family ought to act as decision makers for patients with dementia, as the family’s understanding of the patient’s values becomes less important in the face of the patient’s loss of self.

#### Wider communication

Given the complexity of decisions concerning the initiation of, or withdrawal from, dialysis, it is unsurprising that strong communication arose as a sub-theme. Good communication between all parties is stressed as important, but particularly the central triad of patient, family, and dialysis team [17, 22]. In communicating with families, Brennan and colleagues write, ‘[n]ephrologists should be bilingual; they should speak the plain language of their patients and the technical language of their discipline’ (p. 1006) [13]. This echoes the point made by Keating and colleagues that if a nephrologist does not feel that dialysis is appropriate they should seek to understand the reasons for families wanting continued treatment, as it may be based on a misunderstanding [17].

Communication between professionals is also considered important [12, 16, 20–22, 28]. In their study, however, Scott and colleagues reported variation in how this was reflected in practice, with some interview participants reporting regular multidisciplinary best interests meetings whilst others reported situations in which the decision came down to what the consultant thought best [20]. Some suggest that the involvement of other nephrologists in consultations may be appropriate where disagreement arises that presents a challenge to shared decision making [12], and that a second opinion may even enable patient wishes to be understood more fully [21]. This is considered especially important by Ang and colleagues when the patient has multiple illnesses and is receiving care from doctors of other specialties [22].

Jones and McCullough discuss a particular case in which a vascular surgeon receives a patient referral to establish vascular access for dialysis but does not believe that it is in the best interests of the patient [28]. They argue it would be appropriate in such a scenario for the vascular surgeon to meet with the referring nephrologist to discuss the patient’s care and raise concerns [28]. For Kaye and Lella, the benefit of having input from other professionals, such as nurses and social workers, is the avoidance of a decision being made solely by the patient’s nephrologist [16]. The common thread here is the importance of seeking interprofessional agreement and the presumed benefit of reaching consensus from multiple perspectives; it is considered preferable to involve a broad range of individuals with an interest in the patient’s care and have all parties on the same page regarding the care plan.

Some—notably those situated in the US—specifically discussed the potential role of clinical ethics committees/consultation [12, 15, 17]. Feely and colleagues note that, in their experience, more difficult decisions about dialysis initiation tend to go to an ethics consultation, which they suggest is an appropriate course of action where there is no clear way forward [12]. Similarly, Conneen and colleagues consider ethics consultations a good way of discussing options in a non-adversarial and non-threatening forum—they do, however, stress the importance of documenting deliberations [15].

What the literature demonstrates is some agreement that a collaborative approach is preferable to the subjective opinion of one nephrologist, and that good communication is essential to that.

### Objectivity

Despite the value associated with shared decision making and the importance of individualised decisions, there is a clear concern in the included papers that both nephrologists and families may struggle to approach cases objectively—which indicates that value is placed on objective decision making.

#### Validity of all care options

One sub-theme arising is the importance of all care options being presented in a broadly objective manner, accompanied by appropriate information, thereby allowing the patient (or substitute decision maker, consultee etc.) to make an informed decision (or advise) without undue influence. Foote and colleagues found that some nephrologists dislike the phrase “recommend dialysis”, as a recommendation is not objective [25]. Some note the importance of maintaining a neutral balance by explaining that CKM is not abandonment or opting out of treatment, but is a valid choice [13, 14]. Beyond simply noting that all options are treatment, Spike suggests that nephrologists ought to reassure family that it is both legal and ethical to stop treatment that offers no hope of meaningful recovery [19]. Spike also raises the possibility of enlisting the help of a local hospice to explain alternative options [19].

However, reports of practice in the literature do not align with this idea of the importance of objectivity. CKM is often not raised as an option for patients [13] and, argue Ying and colleagues, social expectations and other pressures have a tendency to lead to overdialysis [31]. A case discussed by Ang and colleagues involved a patient who was eventually persuaded to go for dialysis—the option the nephrologist thought best [22]—suggesting a lack of objectivity in how options were presented. Indeed, some nephrologists have been found to doubt the validity of patient refusals of treatment in the context of ESKD, assuming that a refusal is indicative of psychological problems [21].

There is also a risk of dominant clinician views proving problematic before a patient even reaches the point of dialysis discussion. Campbell and colleagues found that primary care providers were less likely to refer patients with old age and moderate dementia to a nephrologist, with 257/680 accounted for in the study not being referred despite meeting the threshold for referral as per guidelines [32]. This suggests that the reported tendency of nephrologists towards dialysis (noted above) is not shared by primary care providers. Nonetheless, it appears that the line between professional advice (coupled with the objective provision of information) and coercion is an interesting one that might be somewhat blurred at times. As such, Foote and colleagues argue that nephrologists should promote objectivity and consistency by recognizing their treatment preferences and the factors underpinning them [25].

#### Difficulties with family detachment

Family members—and indeed unrelated individuals who are close to patients and might be involved in care decisions—have also been found to struggle with objectively assessing treatment options. Patients often prefer family members to provide input when making decisions [23]. However, as noted above, the assumption that the family will decide in the best interests of the patient may not always be correct [15], and families have been found to make decisions more in their own interests than those of the patient [20]. For example, instances have been reported in the literature of carers preferring patients to receive in-centre haemodialysis as it gives them a rest a few times a week [20], and Spike noted a case in which the clinical team had concerns that a patient’s wife agreed to the withdrawal of dialysis only because she was fed up [33]. Scott and colleagues report a common belief among some nephrologists that families who want aggressive treatment do not fully appreciate the rigours of dialysis, and may be unwilling to accept mortality; some were reported to opine that some families harbour the unrealistic expectation that dialysis will cure all of their loved one’s issues [20].

Brennan and colleagues highlight that patients sometimes make decisions themselves (where they can) on the basis of the perceived needs and wants of their family [13], demonstrating the strength of family influence. Ang and colleagues suggest that it may be hard for family who are the main caregivers not to bring their own judgement into decisions when the caregiver burden and financial expenses are significant [22]. Seconding this, DeCamp asks whether substituted judgement will be pure when the deciding party is the primary caregiver [23]—arguably there would always be some conflict of interest.

### Cultural sensitivity

Another theme arising in the included papers is cultural sensitivity. This is particularly relevant given our earlier discussion of respecting patient autonomy, which does not hold the central importance everywhere that it does in Western cultures. As such, Davison and Holley suggest that ACP and other ‘autonomy respecting’ interventions may not be suitable if self-determination is not important within the patient’s culture [34].

In some cultures, the head of the family assumes the decision-making role [13] and atomistic autonomy is replaced by the idea of the relational self [34]. In a study comparing the role of advance directives between countries, Sehgal and colleagues found Japanese nephrologists to be far less willing to follow an advance directive when the patient’s family disagree with it; willingness of nephrologists to withdraw dialysis in line with an advance directive fell from 88% when the family agreed to 19% when the family disagreed [35]. Sehgal and colleagues posit a possible reason for this to be the greater emphasis on social relatedness in Japan as opposed to the notion of the autonomous self, going on to note that in Japan it is not uncommon for doctors and families to make care decisions on behalf of *competent* patients [35].

For patients whose culture requires the family to be responsible for care, peritoneal dialysis may be preferable as it is an at-home option; Davison and Holley argue that it is important to consider cultural factors in deciding on treatment modality [34]. However, it is highlighted as equally important that no assumptions are made, as patients may have blended cultural perspectives if they have moved from, for example, a non-Western country to a Western country [34]. Overall, then, there is a suggestion that nephrologists ought to be culturally sensitive and open to different values, whilst making no assumptions.

### Medical versus non-medical factors

No two patient cases are entirely alike, and myriad factors may contribute to a conclusion about the best course of action in any given situation. Many such factors are identified in the literature, ranging from family support to survival benefit. Of course, there is also complex interplay between these factors, and various trade-offs that might be considered. As noted by Clement and colleagues, decisions are often a risk–benefit assessment that accounts for both clinical (medical) and social factors (non-medical) [21].

#### Medical factors

The included literature clearly conveys the message that these decisions are not as simple as ESKD necessitating dialysis. The clinical status (including co-morbidities) of patients beyond kidney function is also relevant, and the presence of cognitive impairment itself is felt by some to be relevant to the dialysis decision.

In several of the included papers, it was shown that some participants and authors felt that the presence of cognitive impairment itself was justification for denying dialysis [21, 25]. One participant clearly stated, ‘I don’t think severely demented patients should be dialysed’ (p. 2307) [25]. Spike argues that when a patient has suffered a permanent loss of cognition, the presumption should move from the continuation of life-sustaining treatment to its withdrawal [19]. Davison and Holley are less certain, but suggest that profound neurological impairment might justify the foregoing of dialysis [34]. The reasoning behind this, suggest Conneen and colleagues, is that when a patient is cognitively impaired—dementia being the focus of their paper—dialysis no longer accomplishes the goal of permitting function as a human being, but instead prolongs the dying process [15]—a view echoed by Keating and colleagues [17]. However, Moss and colleagues found there to be little agreement among dialysis unit medical directors as to whether they would continue dialysis for a patient who develops dementia and has no advance directive [27].

The findings of Foote and colleagues demonstrate a higher likelihood of a patient being recommended for dialysis if they have preserved cognition [25], which was similarly found by Munoz Silva and Kjellstrand in the context of permanently unconscious patients [29]. In noting that evidence suggests a typical dementia patient will not get a survival benefit from dialysis, MacPhail and colleagues argue that an individual decision is necessary [14]. They argue that this prevents generalisations impacting on care, as some patients with dementia may get a survival benefit. On individualised decisions for patients with both ESKD and dementia, Ying and colleagues also highlight that generic rules can result in socioeconomic disadvantage, as less educated patients are more likely to be diagnosed with dementia [31].^14^

Survival benefit is generally considered an important factor. MacPhail and colleagues argue the need to consider illness trajectory, noting that older patients are likely to gain only negligible survival benefit [14]. A participant in a study by Foote and colleagues said that one of his general principles is ‘to avoid dialysis in the population > 80’ (p. 2307) [25]. The reason for this view is not made clear, though is likely attributable to consideration around survival benefit. Clement and colleagues also found that prognosis was an important consideration among participants in their study [21]. It is when survival benefit is limited, and the patient has multimorbidity—including dementia—that CKM is most frequently considered [20]. Davison and Holley also consider comorbidities as particularly relevant, arguing that withholding dialysis may be appropriate if the patient has a non-renal terminal condition [34].

Clearly, the presence of comorbidities does impact on how beneficial dialysis can be, and there seems to be widespread support for comorbidities being relevant to dialysis initiation decisions. However, there appears to be disagreement about how influential comorbidities ought to be, and whether cognitive impairment in particular is an appropriate candidate for a blanket exclusion criterion.

#### Non-medical factors

As important as medical factors appear to be in decisions, the literature also demonstrates a consistent appreciation of non-medical factors. Quality of life is widely considered relevant to dialysis initiation decisions [12, 14, 19–21, 24, 25], which reflects an acknowledgement of how burdensome dialysis can be. Foote and colleagues found that nephrologists, when making recommendations for dialysis, were willing to forego 12 months of survival if it would avoid a significant decrease in the patient’s quality of life [25]. However, others highlight how an attitude of “treat what you can” sometimes results in the continuation of treatment despite, for example, severe frailty [12]. Nonetheless, in considering quality of life, MacPhail and colleagues argue that it should be routinely evaluated; the current focus on efficacy in clinical reviews is, they suggest, too narrow [14].

If quality of life is an important factor, the question of how it should be measured and accounted for becomes important. Some appear to automatically associate cognitive impairment with low quality of life. Spike, for example, suggests that those with advanced dementia or a severe and irreversible brain injury get no benefit from dialysis as they are no longer capable of enjoying life [19]. Some participants in Scott and colleagues’ study questioned whether patients with cognitive impairment have sufficient quality of life, especially if they are bed bound and have to attend dialysis sessions on a stretcher [20]. Along similar lines, Kaye and Lella focus on a distinction between biological life and a ‘higher variant’ which is specifically human; ‘‘life’ in the body (i.e. respiration, heart beat, excretion) permanently without awareness, or a minimal ability to relate to other people, is life without the essence of humanity’ (p. 77) [16]. To keep such a patient on dialysis is, they argue, to prolong dying rather than life, which is ‘morally unsound’ [16]. However, MacPhail and colleagues note that quality of life can be very similar for patients on dialysis and those undergoing CKM, with the main difference being that dialysis patients generally spend more time in hospital and are therefore more likely to die there [14]. This is important to consider as whilst quality of life may be comparable more generally between dialysis and CKM, some patients will much prefer to minimise time spent at hospital; in particular, some will not want to die there.

When considering quality of life, it is usually important to engage with the patient to ascertain their own views. However, acknowledging the challenges of involving cognitively impaired patients (specifically those with dementia) in decisions about their own care—as we have discussed above—McDougall suggests one approach may be to consider the interests and preferences of dementia patients generally [24]. Patients living with dementia will not necessarily experience life on dialysis to be of low quality, for example.

There is little support in the literature reviewed for consideration of resources in dialysis decisions. Spike does consider dialyzing permanently cognitively impaired patients a violation of the responsibility to use resources wisely [19], though this view is unique among the papers included. Further, Spike acknowledges that those with less severe dementia sometimes appear happy, so it may not be easy to draw the line [19]. Cady discusses a hypothetical case in which the renal team cite significant use of resources as a reason for not wanting the patient to initiate dialysis, though they still appeal primarily to questions of harm and benefit to the individual patient [36]. She goes on to argue that the distributive justice argument is ‘weak at best’ as such an approach at the patient level ‘undermines the integrity and violates the trust inherent in the physician–patient and/or nurse-patient relationship’ (p. 126) [36]. Conneen and colleagues reject a utilitarian approach to resource allocation in favour of individual best interests [15]. Further, both McDougall and Kaye and Lella argue that nephrologists should ignore wider issues of cost and resource allocation when making decisions for individual patients [16, 24].

The importance of support outside of the clinical setting was highlighted in some papers. Several studies discussed the importance of support systems, with Foote and colleagues noting the importance of family inclination towards dialysis [25]. Scott and colleagues found that some nephrologists even consider family support to be more important to the success of dialysis than age or clinical condition [20]. Of note, however, they highlight that support is also important to the success of CKM [20]. The extent to which support networks might factor into the decision between dialysis and CKM is, then, not clear.

Nonetheless, O’Rourke and colleagues argue that it is appropriate to account for carer burden, including both actual and opportunity costs [37]. This is interesting given our earlier discussion of family dominance in decisions, with relatives sometimes found to have agreed to the patient foregoing dialysis as it would be too burdensome for those relatives. However, equally, we highlighted those who chose to keep a patient alive for their own benefit, something which both Keating and colleagues and McDougall consider inappropriate. Both argue that subjecting a patient to dialysis in order to provide an emotional benefit to the family is unacceptable [17, 24], with Keating and colleagues making a Kantian argument that to do so would be treating that patient as a means to an end [17].

A final non-medical factor that was commonly discussed was patient nonadherence. As this was particularly dominant in the papers, we have decided to discuss it separately in the next section.

### Managing nonadherent patients

There is widespread recognition that nephrologists are under no obligation to provide dialysis where they consider it inappropriate or excessively burdensome [13, 14, 19]. Spike notes the importance of being willing to withdraw treatment when it is no longer beneficial [19]. One possible reason for withdrawing (or, indeed, withholding) care is nonadherence.

As dialysis (specifically in-centre haemodialysis) requires the patient to sit still for an extended period, when a patient becomes agitated and tries to remove needles there is a risk of harm not only to the patient but to anyone else present. In such situations, the option of restraining the patient for the duration of treatment might be considered. O’Dowd and colleagues present a US case in which a patient with fluctuating capacity was dialyzed against his will whilst the care team awaited a court decision about whether continued dialysis was in his best interests [30]. The court later authorised the hospital to take all measures necessary to provide treatment, including sedation [30]. The following passage describes the resulting situation:

‘We ended up deciding that he would be treated against his will which at times involved dragging a kicking, screaming, hitting person, who may have been HIV+, down to dialysis, strapping him down for 4 hours, putting needles in his arms, and dialyzing him. The dialysis staff was not happy about this and it wasn’t clear that it was the best thing or the right thing to do’ (p. 322) [30].

In this case the staff were uncomfortable with the situation and were undecided whether dialysis under physical restraint was best for the patient. In another case (this one in England and Wales), discussed by Grubb, the clinical team sought a declaration from the court that it was in the best interests of a patient who lacked decision-making capacity, and was uncooperative, to not impose haemodialysis [38]. The court agreed, which the author suggests could be because the judge did not consider the regular use of a high degree of force to be in a patient’s best interests. Feely and colleagues also consider a case of a violently resistant patient who, when not on dialysis, clearly expressed a desire to continue with therapy [12]. The clinical team was unsure of the best way to proceed in terms of respecting the patient’s autonomy and ensuring the safety of both the patient and those around him [12]. McDougall notes that a patient removing needles several times during treatment is a clear sign of distress [24]—the implication being that distress is contrary to their interests.

Ying and colleagues argue that where a patient is agitated and restraint becomes necessary to perform dialysis, the restraint should be taken as an additional harm to be considered in the benefit/burden analysis [31]. In addition, Feely and colleagues suggest it is appropriate to consider the safety of others in these situations, and that discontinuation of dialysis can be justified if the risks to others cannot be mitigated [12]. Further, in the context of dialysis trials, Scott and colleagues found that some nephrologists consider that where patient behaviour puts someone at risk it would be appropriate to cease that trial [20]. MacPhail and colleagues note that cooperation is generally considered a prerequisite for dialysis in many guidelines, which becomes relevant with dementia patients as an outpatient dialysis centre is not a dementia friendly environment [14]. Whereas the aforementioned scholars do not go as far as to suggest an automatic ruling out of dialysis where restraint is necessary, Spike does, making the case that the need for restraint is *prima facie* evidence that dialysis is no longer justified [19]. Further, in an earlier paper, Spike discussed a wife who was more strongly in favour of discontinuing her husband’s dialysis when she found out he would have to be sedated for every session [33].

It may be that freedom from restraint is more highly valued than the life extension dialysis affords, from the perspectives of both nephrologists and those close to patients. That being the case, to forcibly dialyze a patient who is severely uncooperative might usually be deemed not to be in their best interests.

### Substituted judgement

Demonstrative of the strong representation of the US context in the literature, there is significant discussion of substituted judgement. There is also some (albeit negligible) discussion of an alternative best interests approach—though it should be noted that such discussions are not necessarily of “best interests” in the context of the MCA 2005, but best interests as an ethical principle. Nonetheless, substituted judgement was the far more dominant theme.

There is certainly evidence of support for the view that, when making a treatment decision for patients lacking capacity, families have a duty to replicate as far as possible the choice that the patient would have made themselves [15, 17]—this is substituted judgement. However, there is disagreement over whether substituted decisions are made properly. It is highlighted by some that surrogate decision makers frequently fail to predict patient preferences correctly [23], and argued that this can undermine the principle of respect for autonomy that underpins substituted judgement approaches [18]. In contrast, Munoz Silva and Kjellstrand suggest that substituted judgement is generally used wisely, as they found little difference between patients who chose to stop dialysis themselves and those who had the decision to stop made for them by another in terms of type of, site of, and time on dialysis [29]. Regardless of whether one considers substituted judgement appropriate, Cady highlights that it may not be reliable because the people to whom the decision would fall may decline to make decisions on behalf of the patient [36].

For substituted judgement to work, McDougall argues that the patient at the time of the decision must be understood to be the same as the patient known by the surrogate decision maker [24]. Patients with dementia, however, suffer ‘discontinuity of self’, so McDougall argues that appropriateness of substituted judgement ought to be questioned—especially where the previous and current interests of the patient conflict [24]. Even without this discontinuity, Perkins suggests that a relative acting as a substitute decision maker may be unsuitable in that role if they do not know the patient well [26].

The importance of individualised decisions is also stressed. MacPhail and colleagues call for this individualised approach in the context of dementia patients to avoid the provision of inappropriate treatments, noting the importance of early, patient-centred discussion of treatment options [14], but their point can certainly be expanded to include any cognitive impairment. In particular, those with fluctuating capacity would stand to benefit, as they are not always unable to make their own care decisions [38].

Whilst it is not widely discussed, a distinction is drawn in the literature between substituted judgement and best interests [22]. McDougall highlights criticism of the patient-focused nature of the best interests approach, as it does not consider morally relevant burden to others [24]. Even if a patient-focused approach is taken, MacPhail and colleagues argue the need to regularly revisit decisions, as what is initially appropriate may become unacceptably burdensome as the patient’s cognitive impairment (dementia is the focus in this paper) progresses [14]. Perkins also seems to touch on the idea of best interests, albeit without labelling it as such, when he notes that ‘[t]he physicians and nurses should interview his [a hypothetical patient] niece, neighbors, bowling teammates, and friends from the senior citizens’ center to learn about Mr. B’s life style, joys, and previously expressed wishes about medical care’ (p. 131) [26].

Substituted judgement is very clearly the main focus of the literature. Very little attention is paid to the alternative of best interests in the context of dialysis decisions. This is not entirely surprising as the majority of the literature is written in the US context, and it spans more than three decades. Nonetheless, shortcomings of both approaches are highlighted.

## Discussion

This scoping review has brought together a range of arguments and evidence pertaining to dialysis decisions made with, for, and on behalf of cognitively impaired adults with or approaching ESKD. What is hugely apparent from the literature is that there is little consensus on any aspect of this topic. In part, this can be attributed to the range of jurisdictions and years covered—it is to be expected that there will be differences across borders and that ideas will change over time.

Whilst patients are at the centre of these decisions, there are many other stakeholders with arguably legitimate interests who would like to, do, and often should contribute to the decision-making process. However, accommodating all legitimate interests is a difficult balancing act. Families are highlighted as often being dominant in these decisions, which is problematic in several ways. It should be noted that the way families are portrayed may not be a fair reflection of all families in these scenarios, but it remains that this idea of dominance is clearly highlighted in the included papers. First, it is suggested that families often fail to accurately represent the patient’s views and preferences, whether intentionally or not [17, 18]. Families also tend to favour dialysis in almost all situations, generally viewing it as a default “safe” option [17, 18]. Sometimes, families may choose to keep the patient alive because doing so is in their own interests (such as wanting to spend more time with them), which is supported by some of the literature [16]. This seems to be based on the consequentialist argument that everyone’s interests matter equally, and so there should be no default assumption that the interests of the patient outweigh those of affected relatives. Family decision makers may also have unrealistic expectations of what dialysis can do for their relative in terms of curing several ills. The role of the family does vary across jurisdictions, and (in this review) appears most significant in the US, which some have suggested is the result of a more litigious and money-oriented health care system [12, 17, 22]. The literature included in this review represents a range of jurisdictions (as detailed earlier in this paper), and the significance placed on the family in decisions in the US is not necessarily a legal issue, but the ethical concerns raised about it are clear. Regardless of any merit one sees in this approach, it is not in keeping with the requirements of the England and Wales’ MCA 2005; the MCA 2005 Code of Practice is clear that what the family wants is not relevant to a best interests decision [39]. Of note, none of the studies highlighting family dominance in decisions originated in the UK. Excluding those that were published prior to the enactment of the MCA 2005, one possible explanation for this is that the law is being well applied and that the role of the family is being limited to reporting the patient’s views and preferences. Another possible explanation is that the kind of research that could expose family dominance in these decisions is not being done in the UK, or that participants in such studies are aware of how they should and should not present accounts of decision making to be consistent with the law. Further research is needed on this.

Cultural variation is also highlighted as a potential reason for dominant involvement of the family. The importance of autonomy is a heavily Western perspective, and some cultures favour less individualistic approaches to care decisions [13, 34]. This raises important questions about how clinicians should navigate complex decisions about medical treatment with, for, and on behalf of patients without decision-making capacity when such patients are from cultural backgrounds that value autonomy differently and have different expectations and norms about the role of family in decision making. The particular risk is that we make wrong assumptions about what a cognitively impaired person would want, based solely on their apparent cultural background. Whether clinicians make cultural assumptions or consult those close to the patient to ascertain pertinent cultural values, a further challenge may arise in simultaneously navigating these cultural values and the requirements of legislation. Considering two different countries where autonomy is generally highly valued, this may be more straightforward in the US, for example, where (for whatever reason) overt family involvement (*ne* dominance) appears more usual, but in England and Wales (in the context of the MCA 2005), even if it is apparent that the patient lacking decision-making capacity holds values that clearly point to the family as favoured decision makers, the law still does not permit the family to act as surrogate decision makers in the absence of a formal proxy appointment. Thus, when the patient’s perceived preferences (which the MCA 2005 says must be considered) do not align with the Act’s assignment of decision-making roles, it becomes very difficult to be sensitive to this particular aspect of cultural difference. There was limited discussion of the complexities of cultural sensitivity in the literature, but this is an important point to consider in a scenario where the family are looked to as representatives of the patient’s own views and preferences.

Overall, it is clear from the literature that families—or, more accurately, those close to the patient—tend to play a significant role in the decision-making process. However, whilst this clinical reality is somewhat reflected in theoretical stances, there is a disconnect. Arguments as to how decisions *should* be made tend to favour a more balanced approach whereby a nephrologist can justifiably question the input of a family and decline to proceed with its choice when, from the perspective of the nephrologist, that choice is not in the best interests of the patient. This supports the MCA 2005′s creation of the consultee role, even if decisions are not always made this way in practice.

Whilst family dominance appears common, there is recognition of the importance of other perspectives. In particular, given how common comorbidities are in the ESKD population, the importance of inter-specialty communication is raised [12, 16, 20–22, 28]. As individuals beyond the renal team will often be involved in the care of a patient with ESKD, there is a suggestion that a decision is better if some sort of consensus is reached. However, according to the literature, this is not always the case in practice [20]. Whilst some clinicians were reported in empirical studies to consider it important to present care options objectively and avoid undue influence [25], in several cases nephrologists appear to doubt patient refusals and consider them indicative of psychological problems [21]. Those in the latter category may consider it acceptable not to take these refusals as informed, autonomous refusals and therefore act paternalistically. In the context of the best interests approach in England and Wales, to treat a patient as lacking decision-making solely on the basis that their decision is not perceived by the clinician as “good”, “right”, or “wise” is contrary to section 1(4) of the MCA 2005 which protects the right of patients to make “unwise” decisions.

A final, and important, related point is that the literature considers ways of avoiding the need for these decisions in the first place. Various forms of ACP are highlighted as a way of respecting the patient’s own wishes [14–19], thereby avoiding potential family dominance and/or paternalism. However, discussion of the pitfalls of such approaches also arises [23]. On a practical note, there is the importance of the level of detail in advance discussions. With dementia, for example, the care preferences of a patient can realistically be expected to be different depending on whether they have mild, moderate, or severe dementia. Distinguishing between these as part of ACP can be challenging, first because the gaps between mild and moderate and moderate and severe may be difficult grasp in advance, and second because ACPs may not contain the necessary level of specificity. Going beyond a discussion of care preferences in the abstract, and considering the patient’s views on treatment in very specific scenarios, can help avoid situations in which recorded patient wishes in relation to future contexts are unclear, but to do so effectively would require such detail so as to be hugely cumbersome. That is before one accounts for the need to keep such plans up to date, and the difficulties raised by the problem of the discontinuous self.

It is perhaps unsurprising that the literature devotes a good deal of space to quality of life, and how it does and ought to factor in decisions about dialysis. This arose in many papers [12, 14, 19–21, 25], though different positions were taken. Whilst some favoured quality of life over quantity (to an extent) [25], others highlighted the presence of a “treat what you can” attitude [12]. Of particular interest, some suggested that cognitive impairment entails a compromised quality of life [16, 19, 20]. Whilst this will certainly be the case for some, it would be wrong to suggest that all cognitively impaired individuals suffer a reduced quality of life or that any reduction in quality on that basis means life is no longer of value. The broad consensus in the literature—to consider the impact on the individual patient rather than make blanket judgements—suggests a broad acceptance of the need to respect autonomy, avoid assumptions, and adopt a patient-centred approach to these decisions.

A further theme we particularly want to highlight is nonadherence. Even if dialysis appears to be clearly in the best interests of a patient, resistance in various forms presents an obstacle to care provision. When a patient is nonadherent, there are risks not only to the patient but to those around them—Hashmi and Moss list examples such as physical abuse and requiring unscheduled extra treatments due to treatment nonadherence [40]. Restraint might seem an obvious remedy, but this is not a course of action to be taken lightly. In one case discussed above, there was regret among the clinical team when a patient was restrained for the purposes of dialysis [30]. There may be a risk of moral distress if clinicians are expected to restrain a patient for dialysis but consider it inappropriate, especially if the restraint is court ordered. Whilst some have suggested cooperation to be a prerequisite of dialysis [14, 19], others are more sympathetic to at least some level of restraint [12, 38]. The extent to which the need for restraint should be factored into dialysis decisions is a moot point in the literature and is complex and often uncomfortable—perhaps because it captures so dramatically the tension between our aversion to coercion and our desire to protect people we see as vulnerable. Given that our aversion to coercion is generally based on acceptance of the importance of respecting autonomy, it may be worth considering whether the discomfort with constraint comes from latent (but perhaps misplaced) feelings that the cognitively impaired patient still has autonomy (which is insulted by coercion) or whether it exposes our concern that the patient still has a right to autonomy that we are consciously or erroneously failing to respect. Whichever—if either—it is, it seems reasonable that the nature of the necessary restraint might affect the extent to which it is considered acceptable or ethically permissible, as well as the frequency and duration of restraint necessitated by the particular patient’s nonadherence.

Especially pertinent to the wider project of which this scoping review is a part is the discussion of the respective merits of the best interests and substituted judgement approaches—notably, that the former is mentioned very little. Some suggest that patient preferences should be the sole guiding force in dialysis decisions [15, 17], but this is problematised by evidence that surrogate decisions often poorly predict patient preferences [23]. This is, as some acknowledge, in part related to the important distinction that can be made between the patient known by the surrogate decision maker and the patient as they are at the time of the decision [24]. The two are not necessarily the same and may be hugely different in terms of how they would view the initiation of dialysis. Perhaps, then, substituted judgement is only appropriate when the two align, but this raises the complex matter of determining whether they do. The difficulty of this might be taken to suggest that the best interests approach is preferable as it aims not to make the decision the patient would actually make, but the best decision that the patient could make. However, this is differently complex and raises challenging questions about the extent to which best interests decisions are intended to track autonomy, act paternalistically, or track some kind of supposed ideal preference. Either way, this review has highlighted a gap in the literature around best interests decisions as described in the MCA and maintenance dialysis.

### Limitations

We acknowledge the potential for bias in this study but accept that this kind of research is always subject to personal interpretation of the material. We sought to minimise bias from JAP’s interpretation by another author (JI) reviewing eligibility decisions and analysis throughout and maintaining a questioning and reflexive attitude towards the themes being developed.

The choice of databases aimed at capturing as much relevant literature as possible. Nonetheless, it may be that some has been missed. Further, by limiting our search to these databases we did not include relevant professional guidance. Nonetheless, such guidance is more akin to the “rules” at work and might not be reflective of the realities of clinical practice, so its inclusion would have necessitated a shift in focus.

This review is exploratory and does not seek to provide a definitive overview, nor does it seek to answer the normative questions that it foregrounds. As such, even accounting for these limitations, this review provides a foundation from which future research concerning the provision of dialysis for cognitively impaired adult patients can build.

## Conclusions

What this scoping review has demonstrated is that there is significant variation in both the practice and theory of dialysis decision making with, for, and on behalf of cognitively impaired adult patients. Decisions made with, for, and on behalf of patients who lack decision-making capacity are almost always challenging but can be more so when care options are as cumbersome as dialysis. Complexity arises in considering who should get a say, how influential their say should be in a decision, and what factors are most relevant to the decision.

This scoping review provides a useful groundwork from which further research can be undertaken, and has highlighted a dearth of literature looking at best interests decisions and dialysis (as per the MCA) and empirical research on these decisions in the UK (and outside of the US generally). This future research might have increasing importance in the era of COVID-19, given the pandemic’s real and potential impact on both kidney care [41] and decision making for the cognitively impaired [42].

## Data Availability

Data sharing is not applicable to this article as no datasets were generated or analysed during the current study.

## Abbreviations

ACP: Advance care planning
CKM: Conservative kidney management
ESKD: End-stage kidney disease
MCA 2005: Mental Capacity Act 2005
UK: United Kingdom
US: United States

## Footnotes

1 Some aspects of the MCA 2005 apply to those aged 16 and 17, but other legislation also applies for such patients—notably, the Children Act 1989.

2 Indeed, Explanatory Note 28 of the MCA 2005 explicitly states that best interests is not a test of substituted judgement, but is an ‘objective test as to what would be in the person’s best interests’.

3 Arksey and O’Malley use the term “studies” rather than “papers”. We specifically use “papers” as it is more inclusive; not all records included in this review are “studies”. Nonetheless, in our later discussion we do refer to studies where appropriate.

4 There is some variation in the MeSH terms used by each database, so this search string was altered slightly when necessary.

5 Some databases did not return results up to the day of the search as they update their records on a weekly basis. The databases which did not return papers published up to and including the day of the search were PsychINFO (up to and including February week 4, 2020) and Embase (up to and including week 9, 2020).

6 One of these three papers - Cady [36] - was not strictly found in the reference list of an already included paper. One of the already included papers was a commentary on it and mentioned it but did not directly reference it.

7 Under this heading, initial thoughts on codes were noted.

8 A Ulysses contract, in this context, is a decision made by a person with decision-making capacity with the intention of binding that person to that decision in future. This is the principle applied to advance directives. The Ulysses contract is based on the story of Ulysses (Odysseus in the original Greek), whereby he foresaw irrational thoughts on approaching the Sirens so instructed his men to prevent him going to them regardless of his later actions and instructions.

9 The dominance of the family in decision making will be discussed shortly.

10 The issue of litigation was raised in the US context, with Feely and colleagues specifically noting a lesser tolerance for dangerous behaviour in the UK [12].

11 It should be noted that such an approach is prevalent in the US but is less so in England and Wales. Certainly, families are consulted, but the practice of best interests is intended as an alternative given the shortcomings of substitute decision making.

12 Again, the differences between countries are highlighted here. All three of these studies originated in the US.

13 This study originated in the US, where the transfer of patients between providers is more common than in England and Wales.

14 They note that tests of cognitive ability look for signs that more educated individuals are more likely to demonstrate.
